# Real-World Management and Outcomes of Crizotinib-Treated ROS1-Rearranged NSCLC: A Retrospective Canadian Cohort

**DOI:** 10.3390/curroncol29030160

**Published:** 2022-03-14

**Authors:** Amanda J. W. Gibson, Adrian Box, Winson Y. Cheung, Michelle L. Dean, Anifat A. Elegbede, Desiree Hao, Aliyah Pabani, Randeep Sangha, Dafydd Gwyn Bebb

**Affiliations:** 1 Department of Oncology, Cumming School of Medicine, University of Calgary, 3330 Hospital Drive NW, Calgary, AB T2N 4N1, Canada; ajwgibso@ucalgary.ca (A.J.W.G.); adrian.box@albertaprecisionlabs.ca (A.B.); winson.cheung@albertahealthservices.ca (W.Y.C.); mlisaac@ucalgary.ca (M.L.D.); anifat.elegbede@albertahealthservices.ca (A.A.E.); desiree.hao@albertahealthservices.ca (D.H.); aliyah.pabani@albertahealthservices.ca (A.P.); 2 Alberta Precision Laboratories, Molecular Pathology Laboratory, 3535 Research Road NW, Calgary, AB T2L 2K8, Canada; 3 Tom Baker Cancer Centre, Alberta Health Services, 1331 29th Street NW, Calgary, AB T2N 4N2, Canada; 4 Faculty of Medicine and Dentistry, University of Alberta, 8440 112 Street, Edmonton, AB T6G 2R7, Canada; randeep.sangha@albertahealthservices.ca; 5 Cross Cancer Institute, Alberta Health Services, 11560 University Avenue, Edmonton, AB T6G 1Z2, Canada

**Keywords:** ROS1-rearranged NSCLC, targeted therapy, real-world outcomes, crizotinib, metastatic disease, biomarker testing

## Abstract

The use, safety and effectiveness of crizotinib as part of the management of ROS1-rearranged NSCLC patients in a real-world Canadian clinical cohort was the focus of this retrospective review. Twenty-one ROS1-rearranged patients with advanced/metastatic disease receiving crizotinib between 2014–2020 were identified; crizotinib demonstrated tolerability and effectiveness in this population where outcomes were similar to those described in other crizotinib-treated real-world cohorts, but lower than those of the PROFILE 1001 clinical trial population. Systemic anti-cancer therapy prior to crizotinib initiation occurred in half of the study cohort, with platin-pemetrexed and immune checkpoint inhibitors being most common. Platin-pemetrexed showed good effectiveness in this cohort, but despite high prevalence of upregulated PD-L1 expression, immune checkpoint inhibitors showed poor effectiveness in his cohort. Among all systemic therapies received, crizotinib showed the most effective disease control, although longer intervals between diagnosis and crizotinib initiation were more common among those showing a lack of clinical response to crizotinib, and patients with brain metastases at the time of crizotinib initiation also showed increased diagnosis to crizotinib initiation intervals and decreased clinical response to crizotinib. This study reveals crizotinib has clinical benefit, but timely identification of ROS1-rearrangements and initiation targeted therapies appears important to maximize outcome in this population.

## 1. Introduction

*ROS1*-rearrangements have been identified as an oncogenic driver in a variety of human solid tumors and is present in 1–2% of cases of non-small cell lung cancer (NSCLC). Inter- and intrachromosomal rearrangements of *ROS1* result in gene fusions which activate the *ROS1* kinase domain facilitating cell division and survival [1]. As ROS1-fusions are both phylogenetically and homologically similar to ALK-fusions, effective *ROS1*-inhibition can be achieved using some pre-existing *ALK*-inhibiting drugs, like crizotinib [2,3]. *ROS1*-rearranged NSCLC represents another genetically unique population of NSCLC amenable to targeted therapy [4].

Although the efficacy of ROS1-inhibitors is well established, the low incidence of *ROS1*-fusions in NSCLC poses evidence gathering challenges. The accepted survival gain associated with crizotinib treatment in *ROS1*-rearranged NSCLC patients is derived mainly from non-randomized phase II clinical trials (Appendix A, Table A1), some of which require three years to accrue as few as 50 patients [5]. It is also recognized that patients seen in routine clinical practice are more diverse than those accrued to clinical trials. A tendency to defer *ROS1*-fusion testing until onset of late stage advanced or metastatic disease, often on an ad hoc basis after ruling out other oncogenic drivers such as *EGFR* and *ALK*, adds another layer of clinical heterogeneity [6]. Recognition of these issues has led to an increased interest in real world evidence to confirm the impact of these new therapies in the general population. Real-world data which accurately captures the inherent variation in patients and clinical scenarios found in everyday clinical practice is uncommon.

To address this need, we conducted a retrospective analysis of the outcome, safety, efficacy and experiences associated with the use of crizotinib in the management of a population-based, real-world clinical population of *ROS1*-rearranged NSCLC within a universal healthcare system, in order to compare outcomes to other global real-world and clinical trial populations. This study used treatment and outcome data on all crizotinib-treated *ROS1*-rearranged patients treated in Alberta, Canada from 2014–2020.

## 2. Materials and Methods

This study used the Glans-Look Lung Cancer Research (GLR) database which captures patient-level demographic, clinical, treatment, response and outcome data via chart reviews of electronic medical records for every patient with a diagnosis of lung cancer within the Canadian province of Alberta (population ~4.4 million). The data in the GLR used for this analysis were collected under ongoing institutional review board approved protocol at our institution (HREBA.CC-16-0574), and as a retrospective review, no patient consent is required. Study data within the GLR database are collected and managed using the Research Electronic Data Capture (REDCap) data capture tools hosted at the University of Calgary [7,8], but are not available for public release.

### 2.1. Patient Selection

Patients were included in this retrospective review if they possessed unresectable locally advanced or metastatic NSCLC harbouring a *ROS1*-fusion, identified according to the International Association for the Study of Lung Cancer/Association for Molecular Pathology/College of American Pathologists biomarker guidelines (2018) [9]. In accordance with these guidelines, *ROS1*-fusion was identified by fluorescence in-situ hybridization (FISH) (from 2014–2018) or through immunohistochemistry (IHC) screening, with cytogenetic FISH confirmation (2019—present). Additionally, for inclusion in this retrospective review, patients must have received crizotinib via the Alberta Health Services central pharmacy dispensing system between January 2014 and June 2020. At the time of this study, only one targeted *ROS1*-inhibitor, crizotinib, was approved for first-line use in Canada (approved for *ROS1*-rearrangements as a first-line targeted therapy by Health Canada in November 2017 [10]; available prior to this time via special access/compassionate use programs, but dispensed through Alberta Health Service pharmacy). Patients receiving crizotinib outside of the centralized Alberta Health Services pharmacy dispensing system (i.e., utilizing a prescription generated outside of Canada, or through crizotinib supplied directly to the patient’s home from a pharmaceutical distributor) are not reflected in this study. PD-L1 expression was assessed using the 22C3 pharmDx assay from Dako.

### 2.2. Clinical Response and Outcome

Survival endpoints including median overall survival following post-advanced/metastatic disease diagnosis (mOS; calculated as time from detection of unresectable or metastatic disease until death) and median progression-free survival (mPFS; calculated as time from crizotinib initiation to detection of progressive disease or death during crizotinib treatment), along with treatment patterns, treatment events, response, and outcomes were calculated using data elements contained in the GLR. For determination of best response and progressive disease—serial and periodic, although not adhering to a standardized schedule—diagnostic imaging reports were compared to a baseline CT scan taken prior to initiation of crizotinib therapy. Response was determined using RECIST v1.1; if actual measurements were not reported within the diagnostic imaging reports, then response was recorded based on the documented opinion of the reviewing radiologist [11]. Best response was considered to the best among overall responses, as per RECIST 1.1, and objective response rate (ORR) and disease control rate (DCR) were determined using best response (ORR: best response of partial or complete response; DCR; best response of stable disease or better). Time to best response and time to progression are therefore impacted by an assessment schedule which may differ between patients, as this is a real-world investigation.

### 2.3. Adverse Event Definitions and Capture

Prevalence and management of adverse events were derived from the GLR which captured adverse events from clinical progress notes, urgent care/emergency room reports, hospital discharge reports, pharmacist contact notes, oncology clinic nursing notes and laboratory testing reports. Adverse events were recorded using Common Terminology Criteria for Adverse Events (CTCAE v. 5.0) codes, descriptors and grades, as standardized and grouped according to Medical Dictionaries for Regulatory Activities (MedDRA) Primary System Organ Class (SOC) terms and hierarchy.

### 2.4. Statistical Methods

Demographic and clinical characteristics of the study cohort were summarized using descriptive statistics and univariate methods, including time-to-event models which were assessed using the Kaplan–Meier approach. A *p*-value < 0.05 was considered a priori as statistically significant. All statistical analysis was performed using Stata Statistics/Data Analysis version 12 [12].

## 3. Results

### 3.1. Patient Characteristics

Twenty-one *ROS1*-rearranged patients treated with crizotinib between 2014–2020 were identified. Of these, 38% of patients were alive and 29% were still undergoing crizotinib therapy at the time of analysis. Demographic and clinical characteristics are summarized in Table 1, and crizotinib treatment details, clinical response and outcome are summarized in Appendix B, Table A2.

### 3.2. Crizotinib Treatment Outcomes

**Figure 1:**
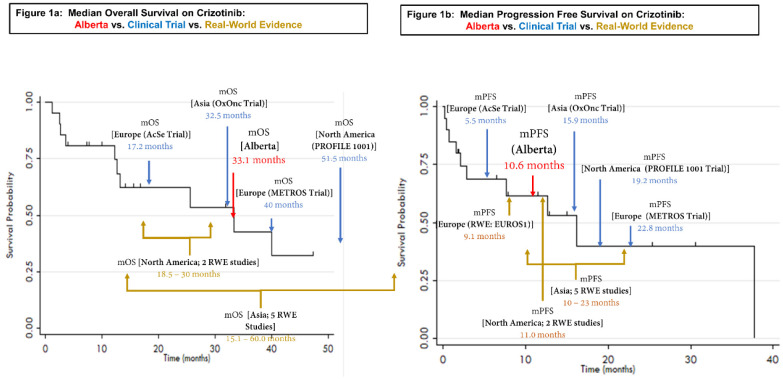
Alberta vs. clinical trial vs. real-world cohorts. Median overall survival on crizotinib (**a**); median progression-free survival on crizotinib (**b**).

Median overall survival (measured from the time of advanced non-resectable/metastatic disease diagnosis), median post-crizotinib overall survival and progression-free survival were 33.1 months, 16.2 months and 10.6 months, respectively (Figure 1a,b presents the comparison to select literature on crizotinib-treated *ROS1*-rearranged NSCLC patients). Patients received crizotinib at a median 58 days (IQR: 29–359) post-diagnosis with advanced non-resectable/metastatic disease, half (52%) receiving crizotinib in the first palliative treatment line. Crizotinib was taken for a median of 6.9 cycles (one cycle = twice daily crizotinib initiation for 21 days; 4.8 months; range: 0–34.2).

**Figure 2:**
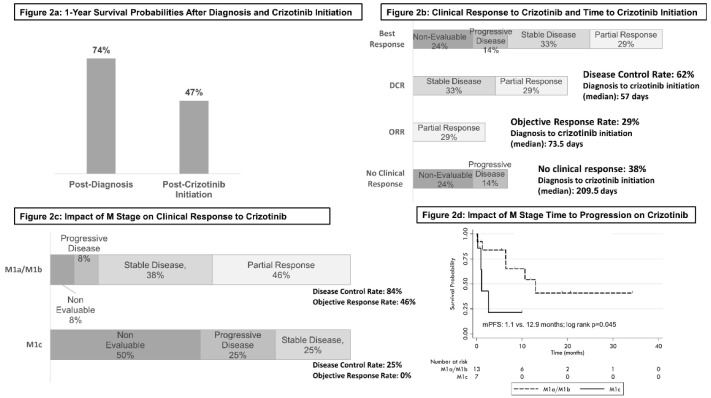
Clinical response to crizotinib: one-year survival (**a**); clinical response to crizotinib and time to crizotinib initiation (**b**); impact of M-stage on clinical response to crizotinib (**c**); impact of M-stage to time to progression on crizotinib (**d**).

The one-year survival rate post-diagnosis was 74%, falling to 47% one year following crizotinib initiation (Figure 2a). Objective response rate (ORR) of 29% and a disease control rate (DCR) of 62% was observed; 38% of the cohort were determined to be non-responders to crizotinib, exhibiting either refractory disease (14%), or terminating crizotinib prior to a measure of clinical response being evaluated (24%). Non-responders showed an increased interval between diagnosis and crizotinib initiation (210 vs. 57 days) (Figure 2b). Median time to best response was 48.5 days, with a median duration of 5.0 months. Higher-order distant metastatic disease (M1c), present in 38% of the cohort at the time of crizotinib initiation, was associated with lower response rates to crizotinib in comparison to those with only intrathoracic (M1a) or single site metastatic disease (M1b): (M1c vs. M1a/M1b: DCR: 25% vs. 84%, *p* = 0.01; ORR: 0% vs 46%, *p* = 0.04), along with lower mPFS (1.1 vs. 12.9 months; *p* = 0.045) (Figure 2c,d). ECOG performance status was not associated with outcome (mOS; mPFS) or response to crizotinib (DCR/ORR).

Treatment discontinuation due to progressive disease accounted for 40% of all crizotinib terminations, primarily extrathoracic progression (86%), most frequently in the brain (57%) and bone (43%); 43% of extrathoracic progression had concurrent intrathoracic progression. Additionally, 33% of crizotinib terminations were a result of death during active treatment, and 20% were a result of adverse events (Appendix B, Table A2).

### 3.3. Adverse Events

In total, 52% of patients reported one or more adverse events (AE) during their crizotinib treatment, at a median 24-days post-crizotinib initiation; most commonly reported AE (CTCAE v5.0) were in the Gastrointestinal Disorders and Investigational Disorders (laboratory values) categories (37% and 32% of all reported AE, respectively). Moreover, 19% of the cohort experienced a serious (grade ≥ 3) AE categorized as Investigational (83%) or Respiratory (17%) Disorders. Elevated transaminases (Investigational Disorders category) were exclusively found in patients of Asian background. In addition, 16% of AE required a treatment break (median 14 days) to allow symptom resolution and two-thirds of treatment breaks saw crizotinib resumed at a reduced dose. All treatment terminations due to AE were in conjunction with severe (grade 3 or 4) adverse events, but the majority of toxicity-related crizotinib terminations went on to receive additional lines of systemic therapy (Appendix B, Table A2).

### 3.4. Non-Crizotinib Systemic Therapies

In total, 24% of patients terminating crizotinib (for any reason) went on to receive other forms of palliative-intent systemic therapy; as nearly one third of crizotinib terminations were due to death, the rate of additional lines of systemic therapy was 63% for those patients surviving > 30 days after crizotinib termination. The predominant subsequent systemic treatment was newer-generation *ROS1*-inhibiting targeted drugs, with all patients accessing additional post-crizotinib systemic therapy receiving one or more additional *ROS1*-inhibitors. Duration of use for the subsequent (range: 1–3) post-crizotinib treatment lines was from 1.9–4.6 months at the time of analysis. Additionally, 24% of the cohort received lorlatinib at some point following termination of their crizotinib therapy; median progression free survival for lorlatinib-treatment was not reached at the time of analysis, with a DCR of 60% (ORR: 20%). As a heavily pre-treated population (57% receiving some form of previous systemic therapy), exposure to platinum-pemetrexed and immune checkpoint inhibitors (ICI) were also investigated: 29% of patients in the cohort received platinum-pemetrexed cytotoxic chemotherapy, predominantly prior to crizotinib (86%), yielding a mPFS of 9.8 months, and DCR of 57%, but pemetrexed exposure did not significantly alter overall survival times. In addition, 57% of the cohort expressed PD-L1, with 48% PD-L1 high (≥50%); demographic and clinical characteristics did not differ significantly by level of PD-L1 expression. ICI was used in 40% of PD-L1 high expressors, and in two patients with PD-L1 negative expression as part of a concurrent chemoimmunotherapy regimen, all prior to crizotinib initiation. Immunotherapy use demonstrated a DCR of 33%, ORR of 17% and mPFS of 10.1 weeks, and 33% of ICI treated patients primarily showed resistance to immunotherapy (Table 2).

### 3.5. Brain Metastases

**Figure 3:**
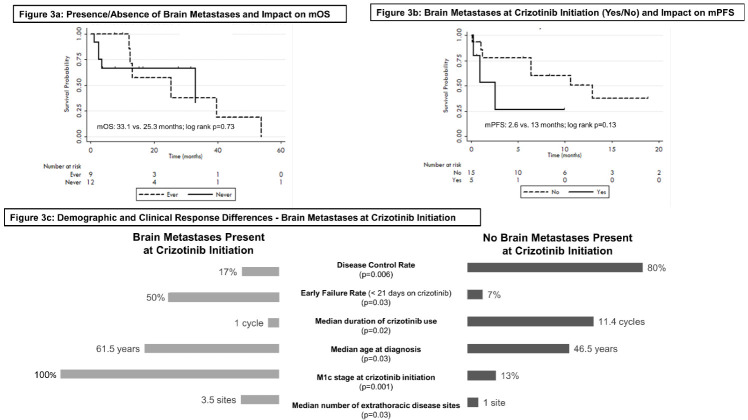
Brain metastases and crizotinib: presence/absence of brain metastases and impact on mOS (**a**); impact of brain metastasis at time of crizotinib initiation and impact on mPFS (**b**); demographic and clinical response differences of patients with/without brain metastases at crizotinib initiation (**c**).

43% of patients had brain metastases at the time of analysis: 29% at the time of crizotinib initiation, and 14% developing brain metastases on crizotinib. Median time to brain metastases development on crizotinib was 6.4 months. Of the patients with brain metastases (either before or during crizotinib), 67% received radiotherapy, predominantly whole-brain radiotherapy (67%). Presence/absence of brain metastases did not significantly impact overall survival (33.1 vs. 25.3 months, *p* = 0.73) (Figure 3a). Presence of brain metastases crizotinib initiation (baseline brain metastases, bBM) did not have a significant impact on mPFS (2.6 vs. 13 months, *p* = 0.13) (Figure 3b), but did result in much lower clinical efficacy than in those without bBM (DCR: 17% vs. 80%; *p* = 0.006). In comparison to those without bBM, those with bBM experienced a higher rate of early failure and death during crizotinib treatment, a significantly shorter duration of treatment (median duration: 1 vs. 11.4 cycles, *p* = 0.02), were older (61.5 vs. 46.5 years, *p* = 0.03), possessed significantly more extrathoracic disease at crizotinib initiation (median: 3.5 vs. 1 extrathoracic metastatic site, *p* = 0.03), and received crizotinib much later following their NSCLC diagnosis (379 vs. 52 days, *p* = 0.08) (Figure 3c).

## 4. Discussion

While randomized clinical trials generate the highest level of clinical evidence, real-world data offer additional insight that contextualize the impact of new practice in routine care [13]. Within an evolving framework, real-world data is increasingly used by regulatory bodies to review new and existing indications [14]. This study investigated the real-world clinical experience of a *ROS1*-rearranged NSCLC cohort on first exposure to a ROS1-inhibiting therapy, crizotinib, in Alberta, Canada. Comprised of 21 patients, this cohort included all patients in the province with a diagnosis of *ROS1*-rearranged lung cancer and treated with one or more doses crizotinib during a six-year period. As such, this cohort mirrors the diversity of demographic characteristics, clinical features, and previous disease management decisions present in real clinical populations. Despite the limitations associated with retrospective analyses, our study makes a rigorous and valuable contribution to our understanding of managing *ROS1*-positive NSCLC [15].

Our real-world study population demonstrated a 33.1-month mOS and median PFS of 10.6 months, meeting or exceeding that of other North American real-world cohorts [2,16,17], and falling mid-range of Asian real-world [18,19,20,21,22] and global clinical trial populations [23,24,25,26,27] (Figure 1a,b; Appendix A, Table A1). While these various real-world cohorts may differ in ways which meaningfully impact outcome, highly variable survival times and the propensity towards reduced median overall survival times, particularly among North American real-world cohorts, should be a cause of concern given that *ROS1*-rearranged lung cancer afflicts younger patients and translates into a significantly reduced life expectancy.

Crizotinib exposure was observed to rapidly produce a modest objective response rate (ORR: 29%; DCR: 62%). This is markedly lower than the >65% ORR observed in recent clinical trials investigating crizotinib [23,24,25,27], and among Asian real-world cohorts [18,19,20,21,22], but the literature is lacking ORR data for comparable North American real-world cohorts (Appendix A, Table A1). Further, this study included all crizotinib-treated *ROS1*-rearranged patients and did not exclude from analysis those patients with short durations of crizotinib treatment, or those without response assessment, as may have been the case in many other clinical trial and real-world reviews. Limiting calculation of DCR and ORR to just those patients with response assessment, a revised estimate of degree of response among those with response data is 81% and 38%, respectively. Within this study, and perhaps afflicting other real-world cohorts, was a high proportion of patients (38%) with multiple sites of distant disease (AJCC 8th edition, M1c stage) at the time of crizotinib initiation. This has been observed among other crizotinib-treated real-world *ROS1*-rearranged populations, underscoring the known poor prognosis of patients with multiple extrathoracic metastases [28], which may reflect a reduced impact of crizotinib on widely disseminated disease [22], and clarifies the contributing factors to the discrepancy between crizotinib-mediated DCR in the literature (range: 80–94%) and that observed in this cohort (62%), as 50% of patients with multiple sites of distant disease also discontinued crizotinib prior to response assessment.

This real-world cohort was characterized by a notable rate of systemic therapy received prior to crizotinib initiation (47%), in the form of immune checkpoint inhibitors and platinum-pemetrexed, but with only a small proportion (24%) receiving additional post-crizotinib systemic therapy upon crizotinib termination. Non-crizotinib systemic therapies yielded mixed results within this cohort. Despite a high rate of PD-L1 expression reflecting a known association between *ROS1*-rearrangements and upregulated PD-L1 [29], response to and effectiveness of ICI in this cohort was limited, with outcomes significantly below those observed in similarly treated, but driver-mutation negative KEYNOTE-42 and 189 cohorts [30,31]. These findings support an emerging body of literature suggesting that ICI use—particularly ICI monotherapy—in *ROS1*-rearranged NSCLC may elicit an inconsistent and generally poor response [32], implying that PD-L1 expression alone may not be sufficient to predict response to ICI, especially in the presence of a driver mutation [33,34]. In contrast, platinum-pemetrexed therapy showed good effectiveness, reflecting a known favorable response to pemetrexed exposure in *ROS1*-rearranged NSCLC [21,35], postulated to be a result of increased pemetrexed sensitivity due to lower expression of thymidylate synthase among the *ROS1*-rearranged genotype [21,36]. Use of lorlatinib, received by every patient in this cohort accessing post-crizotinib therapy, mirrors the disease control rate found in other real-world cohorts [37], and may be key in increasing survival outcomes of contemporary *ROS1*-rearranged patients, particularly those with intra-cranial disease [38], alongside other *ROS1*-inhibitors including ceritinib, entrectinib and repotrectinib [3,22,39]. These findings reinforce the utility of pemetrexed as an effective treatment option in the context of failure or unavailability of targeted *ROS1*-inhibitors [40], and insinuates that the overall survival duration seen within this study cohort is a function of both non-targeted (pemetrexed) and targeted (crizotinib, ceritinib, loratinib) therapies.

Identified in the study is a subset of patients (38%) which was unresponsive to crizotinib; notably, these non-responders also had longer median diagnosis to targeted therapy initiation intervals (209.5 vs. 58 days) than the cohort as a whole. Non-responders were comprised of those exhibiting primary resistance to crizotinib (14% of cohort with progressive disease as best response), a rate complementing the rates of refractory disease (6–14%) seen among other *ROS1*-rearranged populations [5,22,24,41], postulated to be due to co-occurring mutations activating bypass mechanisms, or *Bim* deletion polymorphism [42,43,44]. The remaining 24% were without response evaluation due to crizotinib discontinuation prior to response assessment, primarily due to death during active treatment prior to any response assessment. This probably reflects crizotinib use in the context of poor clinical condition, extensive disease burden and end of life due to its oral-availability and excellent tolerability. Early death after crizotinib initiation in this context is one contributor to the decreased response rates and outcomes of crizotinib-treated *ROS1*-rearranged real-world patients found in inclusive population-level studies such as this one.

In addition, exhibiting a longer interval between diagnosis and crizotinib initiation were those with brain metastases at the time of crizotinib initiation (median 12.5 vs. 1.7 months post-diagnosis). Those with brain metastases at crizotinib initiation had decreased time to progression and significantly reduced duration of use, and response to, crizotinib therapy, a pattern also observed within Phase II clinical trial studies [24,25]. Further, patients with brain metastases at the time of crizotinib initiation were distinct from those developing brain metastases during crizotinib treatment in terms of age and burden of metastatic disease; inferior outcomes among this subset of patients reflect known associations between poorer prognosis among older patients with brain metastases, and the inverse relationship between number of extrathoracic metastatic sites and prognosis [45,46,47]. The brain was also the most common site of failure during crizotinib therapy, reflecting a known inability of crizotinib to penetrate the blood–brain barrier and both prevent and control intra-cranial disease [48]. Brain metastases (at crizotinib initiation or developing during crizotinib) were primarily managed with whole-brain radiotherapy, recognized as an effective management strategy for brain metastases in *ROS1*-rearranged disease [48]. Crizotinib-mediated extra-cranial disease control in those developing brain metastases on crizotinib resulted in better outcomes than those reported in the literature for patients treated solely with radiotherapy to the brain [49], or cytotoxic systemic therapy [50], reinforcing the role of crizotinib in providing an interval of systemic control in conjunction to radiotherapy-mediated intra-cranial control which makes a positive contribution towards patient outcome. Moreover, of note, patients with brain metastases who accessed subsequent post-crizotinib systemic treatment (all receiving lorlatinib) had no further documentation of progression of their intracranial disease to date, findings which signal the efficacy of newer generation *ROS1*-inhibitors to control intracranial disease and improve outcomes [39,51].

This study was able to contextualize the challenge confronting real-world populations after the discovery of a new actionable driver mutation. Exemplified here in *ROS1*-rearranged populations, but applicable to all new instances of actionable mutations, are the barriers in accessing the new companion diagnostic and associated novel targeted therapy [52,53]. In turn, this barrier to access may contribute to the development of clinical features (high order metastatic disease, brain metastases) or necessitate clinical decisions (use of non-targeted systemic therapies, targeted therapy initiation in final days of life) that may—and in the case of this *ROS1*-rearranged population, do—negatively impact outcome. Prior to establishing a routine/reflexive, integrated, in-house testing platform, the increased time to request and perform testing for novel actionable mutations increases the time between diagnosis and targeted therapy initiation; in turn, features generally unfavorable to good response and outcome may develop. The ensuing delays resulted in a median 58-day interval between NSCLC diagnosis and crizotinib initiation within this ROS1-rearranged cohort, exceeding the proposed 42-day diagnosis to systemic therapy initiation benchmarks [54,55]. In addition, a number of studies exclude patients with short duration of crizotinib use and those lacking response assessments, leading to a knowledge gap in regard to the clinical features and outcome of these patients.

This study has some limitations: first, the intrinsic limitations of retrospective reviews, replete with non-standardized response assessment, toxicity reporting and variable follow-up intervals; and second, the small size of this *ROS1*-rearranged population due to the relative rarity of *ROS1*-rearrangements within NSCLC populations. Despite these limitations, this study has several strengths. First, it is a population-based study, and as such represents all Alberta *ROS1*-rearranged patients treated with crizotinib within a regional population of 4.4 million [56]. Second, Alberta possesses a single payer universal healthcare program which diminishes potential disparities in care and treatment access due to financial situation or insurance provider. Finally, comprehensive and fully inclusive real-world data sets within a single healthcare system that eliminate sources of potential collection bias are rare, particularly within the North American context.

## 5. Conclusions

In conclusion, this study, demonstrates the clinical benefit of crizotinib even within a heterogeneous real-world clinical population, and as such complements the findings of clinical trials and other real-world reviews. This study’s findings reinforce the utility of real-world evidence in juxtaposition to clinical trial data and highlights the challenges among some clinical populations in some jurisdictions to timely and funded access to both *ROS1* testing and *ROS1*-inhibitors. Facilitating access to both *ROS1* testing and *ROS1*-inhibiting therapies will serve to increase the survival outcomes possible for this genetically unique NSCLC population.

## Figures and Tables

**Table 1:** Demographic and clinical features for crizotinib-treated ROS1-rearranged cohort.

Demographic or Clinical Feature	Total Cohort (*n* = 21)	
	*n* (%)	
Age at treatment initiation		
Median (years), (IQR)	51.6 (43.9–59.7)	
<50 years	10	
≥50 years	11	
Sex		
Male	7	
Female	14	
Smoking Status		
Never Smoker	18	
Ever Smoker	3	
Body Mass Index (kg/m^2^)		
Median, (IQR)	23.6 (22.6–26.7)	
<18.5 (underweight)	0	
18.5–24.8 (normal)	10	
24.9–29.9 (overweight)	6	
>29.9 (obese)	1	
Missing data	4	
Race		
Asian	8	
Caucasian	12	
Non-Asian/Non-Caucasian	1	
Geographic Location of Residence		
Urban	21	
Rural	0	
Cancer Treatment Centre Type		
Academic	20	
Community/Regional	1	
ROS1 Testing		
Testing Location:		
Within Canada	5	
Outside Canada (USA; Germany)	16	
Testing Funding:		
Provincial Health System Funding	7	
Patient Funded	7	
Unknown	7	
Time from Diagnosis to Crizotinib Initiation		
Median (IQR)	58 days (29–359)	
Eastern Cooperative Oncology Group Performance Status	At Diagnosis	At Crizotinib Initiation
Good (0 or 1)	16	14
Poor (2 or 3)	4	6
Unknown	1	1
Histological Subtype		
Adenocarcinoma	21	
PD-L1 Status (at diagnosis)		
Negative (<1%)	3	
Low (1–49%)	2	
High (≥50%)	10	
Not tested/insufficient sample	6	
Metastatic Disease Presentation		
Upon Relapse	2	
(following resection for early stage disease)		
Advanced/Non-resectable Disease at diagnosis	1	
De novo Stage IV	18	
(metastatic disease present at diagnosis)		
Previous Systemic Therapy Exposure		
Curative-intent (adjuvant cytotoxic chemotherapy)	2	
Palliative-intent	10	
(cytotoxic chemotherapy or immune checkpoint inhibitors)		
None (treatment naive)	9	
Previous Thoracic Radiation Therapy Exposure		
None	10	
Curative to thorax (>4500 cGY)	2	
Palliative to Thorax	9	
Brain Metastases Development		
None (to date)	12	
At baseline	6	
During crizotinib therapy	3	

IQR: interquartile range.

**Table 2:** Additional non-crizotinib systemic therapies: outcome and effectiveness.

	Platin-Pemetrexed (+/−Maintenance Pemetrexed Therapy) (*n* = 7)		Lorlatinib (*n* = 5)		Immune Checkpoint Inhibitors (*n* = 6)	
Treatment Sequence						
Prior to Crizotinib	6 (86%)		0 (0%)		6 (100%)	
After Crizotinib	1 (14%)		5 (100%)		0 (0%)	
Duration of Treatment						
Median	7.8 months		118 days		33.5 days	
IQR	21 days–14.3 months		16–140 days		21–231 days	
Range	18 days–5.5 years		11–845 days		1–294 days	
Best Response						
Complete Response	0 (0%)		0 (0%)		0 (0%)	
Partial Response	0 (0%)	ORR: 0%	1 (20%)	ORR: 20%	1 (17%)	ORR: 17%
Stable Disease	4 (57%)	DCR: 57%	2 (40%)	DCR: 60%	1 (17%)	DCR: 33%
Progressive Disease	1 (14%)		0 (0%)		2 (33%)	
Non-evaluable	2 (29%)		2 (40%)		2 (33%)	
Progression-Free Survival						
Median [95% CI]	9.8 months [0.2–NR]		NR [16 days–NR]		10.1 weeks [23 days–NR]	
Overall Survival						
(post-metastatic disease discovery)						
Median [95% CI]	39.6 months [12.1–NR]		39.6 months [13.1–NR]		12.5 months [2.6–NR]	

IQR: interquartile range; ORR: objective response rate; DCR: disease control rate; NR: not reached.

## Data Availability

The data presented in this study may be available on request from Alberta Health services, as this data is not publicly available.

## Appendix A

**Table A1:** Overview of outcomes for clinical trial and real-world studies with crizotinib-treated ROS1-rearranged non-small cell lung cancer (NSCLC).

Study	Study Type	Median Progression-Free Survival [95% CI]	Median Overall Survival [95%CI]	ORR	DCR
Li et al., 2018	Real-World Evidence (China)	12.6 months [IQR: 7.7–19.3]	32.7 months [IQR: 18.8–NR]	83.3%	97.2%
Liu et al., 2019	Real-World Evidence (China)	11.0 months [7.8–14.2]	41 months [22.5–59.5]	71.5%	94.3%
Masuda et al., 2019	Real-World Evidence (Japan)	10 months [5.1–27.0]	28.7 months [6.7–NR]	90%	
Park et al., 2018	Real-World Evidence (Korea)	13.1 months [4.4–NR]	15.1 months [5.4–NR]	73.3%	80.1%
Zheng et al., 2020	Real-World Evidence (China)	23.0 months [22.4–33.6]	60.0 months [40.7–79.3]	64.7%	94.1%
Doebele et al., 2019	Real-World Evidence (USA Flatiron Health Dataset)	8.8 months [8.2–9.9] (time to treatment discontinuation)	18.5 months [15.1–19.9]	-	-
Gainor et al., 2017	Real-World Evidence (USA)	11.0 months	30 months [12–NR]	-	-
Mazieres et al., 2015	Real-World Evidence (Europe: EUROS1)	9.1 months	-	80%	86.7%
Patil et al., 2018	Real-World Evidence (USA)	11.0 months [8.0–23.0]	-	-	-
PROFILE 1001 (as reported by Shaw et al., 2019)	Phase I (NCT00585195)	19.3 months [15.2–39.1]	51.5 months [29.3–NR]	72%	91%
OxOnc (as reported by Wu et al., 2018)	Phase II (NCT01945021)	15.8 months [12.9–24.0]	32.5 months [32.5–NR]	71.7%	-
EUCROSS (as reported by Michels et al., 2019)	Phase II (NCT02183870)	20.0 months [10.1–NR]	Not reached (data immature) [17.7 months–NR]	70%	90%
AcSé (as reported by Moro-Silbot et al., 2019)	Phase II (NCT02034981)	5.5 months [4.2–9.1]	17.2 months [6.8–32.8]	47.2%	-
METROS (as reported by Landi et al., 2019)	Phase II (NCT02499614)	22.8 months [15.2–30.3]	40.0	65%	85%

## Appendix B

**Table A2:** Clinical response and outcome for ROS1-rearranged NSCLC on crizotinib.

Treatment Details, Response and Outcome	Total Cohort (*n* = 21)	
	*n* (%)	
One Year Survival Rate [95% CI]		
After Diagnosis	74% [48.9–88.4]	
After Crizotinib Initiation	47% [24.4–67.2]	
Five Year Survival Rate [95% CI]		
After Diagnosis	11% [0.7–37.1]	
After Crizotinib Initiation	0%	
Median time on crizotinib (net duration of use)		
Cycles, (IQR)	6.9 (1.3–17.1)	
Systemic Treatment Line in which crizotinib was received		
1st Palliative Line	11	
2nd Palliative Line	8	
3rd Palliative Line	0	
4th Palliative Line	2	
Median, (IQR)	1 (1–2)	
Best Response Metrics		
Complete response	0	
Partial response	6	
Stable disease	7	
Progressive disease	3	
Non-evaluable	2	
Time to best response (days), (IQR)	48.5 (32–65.5)	
Duration of best response (months), (IQR)	5.0 (0.3–10.8)	
Disease Control Rate	62%	
Objective Response Rate	29%	
Adverse Event(s) Occurred		
No	10	
Yes	11	
Adverse Event Management (*n* = 19 adverse events)	% of all AE	% of cohort
No treatment modifications	11	15
Terminate crizotinib	5	3
Treatment break only	1	1
Dose modification only	0	0
Treatment break and dose modification	2	2
Median duration of treatment break, (IQR)	14 days (14–153)	
Dose modification	250 mg twice daily reduced to 200 mg twice daily	
Highest Grade of Reported AE, per patient		
None	10	
Low (grade 1 or 2)	7	
Serious (grade 3 or 4)	4	
Number of Adverse Events per Patient		
Median, (IQR)	1 (0–2)	
Time to First Reported Adverse Event (days)		
Median, (IQR)	24 (7–35)	
Adverse Events Reported (CTCAE 5.0 Category)		
Eye Disorders: Floaters		
Yes	2	
No	19	
General Disorders: Edema		
Yes	1	
No	20	
Gastrointestinal Disorders:		
Nausea, Vomiting, Diarrhea, Constipation, Abdominal pain		
Yes	6	
No	15	
Investigational Disorders:		
Increased ALT, AST or liver function test		
Yes	3	
No	18	
Nervous System Disorders: Dizziness		
Yes	1	
No	20	
Respiratory System Disorders: Pneumonitis		
Yes	1	
No	20	
Reasons for crizotinib termination	% of terminations	
Progressive Disease	6	
Adverse Events	3	
Death	5	
Different Treatment Option Identified	1	
Additional Systemic Therapy post-crizotinib		
Yes	5	
No	10	
Crizotinib ongoing	6	
